# Cognitive flexibility predicts early reading skills

**DOI:** 10.3389/fpsyg.2014.00565

**Published:** 2014-06-11

**Authors:** Pascale Colé, Lynne G. Duncan, Agnès Blaye

**Affiliations:** ^1^Laboratoire de Psychologie Cognitive, UMR-7290, Aix-Marseille UniversityMarseille, France; ^2^School of Psychology, University of DundeeDundee, UK

**Keywords:** reading acquisition, reading comprehension, cognitive flexibility, semantics, phonology, executive function

## Abstract

An important aspect of learning to read is efficiency in accessing different kinds of linguistic information (orthographic, phonological, and semantic) about written words. The present study investigates whether, in addition to the integrity of such linguistic skills, early progress in reading may require a degree of cognitive flexibility in order to manage the coordination of this information effectively. Our study will look for evidence of a link between flexibility and both word reading and passage reading comprehension, and examine whether any such link involves domain-general or reading-specific flexibility. As the only previous support for a predictive relationship between flexibility and early reading comes from studies of reading comprehension in the opaque English orthography, another possibility is that this relationship may be largely orthography-dependent, only coming into play when mappings between representations are complex and polyvalent. To investigate these questions, 60 second-graders learning to read the more transparent French orthography were presented with two multiple classification tasks involving reading-specific cognitive flexibility (based on words) and non-specific flexibility (based on pictures). Reading skills were assessed by word reading, pseudo-word decoding, and passage reading comprehension measures. Flexibility was found to contribute significant unique variance to passage reading comprehension even in the less opaque French orthography. More interestingly, the data also show that flexibility is critical in accounting for one of the core components of reading comprehension, namely, the reading of words in isolation. Finally, the results constrain the debate over whether flexibility has to be reading-specific to be critically involved in reading.

## INTRODUCTION

Reading acquisition has mainly been investigated from a psycholinguistic perspective which has been instrumental in identifying the important developmental impact of linguistic skills such as phonological awareness (Harm and Seidenberg, 1999; Ziegler and Goswami, 2005; Sprenger-Charolles et al., 2006). However, reading can also be viewed as a complex cognitive task, which requires the capacity for the concurrent processing of multiple aspects of print, and which, as a result, may implicate more general cognitive processes, such as executive function (Cartwright, 2002, 2012; van der Sluis et al., 2007).

Executive function (EF) serves as an umbrella term for the control functions that monitor the cognitive processing involved in complex, goal-oriented tasks (Miyake et al., 2000; Best and Miller, 2010). The “unity and diversity” view of EF (Miyake et al., 2000; Miyake and Friedman, 2012), emphasizes a common underlying ability to maintain task goals (unity), together with three distinguishable components (diversity), namely shifting of mental sets, inhibition of prepotent responses and updating of working memory representations.

The focus of the present study will be on shifting, also described as cognitive flexibility. This refers to the ability to select adaptively among multiple representations of an object, perspectives or strategies in order to adjust to the demands of a situation (Chevalier and Blaye, 2009; Cragg and Chevalier, 2012; Diamond, 2013). Cognitive flexibility is involved in the acquisition of theory of mind (Müller et al., 2005) but it is the role that flexibility is thought to play in academic learning skills (Bull and Scerif, 2001; Bull et al., 2008; Yeniad et al., 2013)^1^ that has led to our focus on this aspect of EF in relation to reading acquisition. At present, evidence for a direct link to reading is mixed – although several studies that are largely restricted to the English language have supported a positive association between flexibility and reading (Cartwright, 2002, 2007; Cartwright et al., 2010; Kieffer et al., 2013), other studies have failed to find such a relationship among typical or disabled readers of Dutch and French (van der Sluis et al., 2004, 2007; Monette et al., 2011). The differences between these outcomes will be explored in the sections to follow by examining the tasks used, the type of reading skill and the domain specificity of flexibility skills.

Cognitive flexibility is most often examined using task-switching paradigms, measuring the ease of switching between different sets of sorting rules, which reveal initial successes between the ages of 3 and 5 years (Cragg and Chevalier, 2012), and from 7 to 9 years, an increasing capacity to deal with multiple dimensions in switching tasks (Anderson, 2002). The relatively late emergence of flexibility in task switching has been attributed to partial dependence on other EFs (Davidson et al., 2006; Garon et al., 2008). Authors have variously emphasized the underlying role of: (1) inhibition, either the inhibition of the previous rule (Kirkham et al., 2003) or the disinhibition of the previously inhibited sorting rule (Müller et al., 2006; Chevalier and Blaye, 2008); and (2) working memory, as part of goal setting and maintenance (Marcovitch et al., 2007).

Other measures of flexibility such as fluency in producing multiple uses for a single object (Diamond, 2013) and matrix classification tasks (e.g., Piaget and Inhelder, 1958), reveal a more specific aspect of flexibility, which is conceptualized theoretically as the difficulty in processing two or more dimensions simultaneously. In the revised Cognitive Complexity and Control model (Zelazo et al., 2003), the processing of dimensions simultaneously is regarded as more complex than switching between dimensions and is thought to be constrained developmentally by the conscious (meta-cognitive) control required (Zelazo, 2004)^2^.

Finally, it is evident that considerable overlap exists between cognitive flexibility and the Piagetian concept of decentration in concrete operational thinking (Miller, 2010), since both depend on the ability to focus on more than one dimension of a problem. This comparison with the more intensively researched Piagetian concept highlights interesting questions, in particular, whether flexibility can be considered to be domain-general versus domain specific, a question to which we return in our experimental work. An initial investigation of this question suggests that EF skills do not generalize between verbal and non-verbal stimuli, at least among the kindergartners studied (Foy and Mann, 2013).

Several authors have presented a case for the involvement of cognitive flexibility in the development of reading and reading-related skills. The emergence of meta-linguistic awareness, a key component of beginning reading, has been linked to concrete operational thinking, which shares features with cognitive flexibility (as discussed above). Meta-linguistic awareness entails the switching of attention from word meaning to consider other properties of language such as phonology. Tunmer et al. (1988) reported that Grade 1 phonological awareness was partly dependent on level of operativity in tasks such as matrix classification and class inclusion. More recently, Blair and Razza (2007) used an item-selection task (Jacques and Zelazo, 2001), requiring item representation along two dimensions, to reveal correlations between flexibility and both phonological awareness and letter knowledge among kindergartners. Pre-school associations have also been found between flexibility (Dimensional Change Card Sort task) and emergent literacy skills such as phonological and print awareness (Bierman et al., 2008), as well as between theory of mind (Unexpected Location/Contents and Mistaken Identity tasks), flexibility (Wisconsin Card Sorting task), and rhyming skill (Farrar and Ashwell, 2012).

Flexibility, as measured by matrix classification, has also been found to correlate directly with early word reading and reading comprehension (Arlin, 1981; Hogan and Whitson, 1984). Berninger and Nagy (2008) account for such findings by proposing that flexibility may be required to establish cross-modal connections between spoken and written language and to acquire and coordinate multiple features of print (phonology, morphology, syntax, semantics) during the development of word recognition. If so, flexibility may also underpin reading comprehension which is thought to be the product of word recognition and oral language comprehension (Simple View of Reading, Gough and Tunmer, 1986; Tunmer and Chapman, 2013). Cartwright (2002, 2007) has further argued that cognitive flexibility will play an even more direct role in reading comprehension due to the requirement to process phonological codes for written word recognition simultaneously with the semantic information for comprehension.

Cartwright (2002) provided evidence for this latter claim by studying the cognitive flexibility of English-speaking second to fourth graders in relation to their reading comprehension. A general flexibility task (Bigler and Liben, 1992) was administered, requiring double classification of sets of line drawings of objects into a 2 × 2 matrix using visual (same color) and semantic (same superordinate category) dimensions simultaneously. Cartwright also examined a form of reading-specific flexibility, which involved classification of written words into a 2 × 2 matrix according to phonological (same initial phoneme) and semantic (same superordinate category) criteria. The results indicated that reading-specific flexibility contributed unique variance to reading comprehension beyond the (significant) contributions of age, general flexibility, pseudo-word naming and oral language comprehension. A second experiment, demonstrated that a group receiving a short training in reading-specific flexibility using the matrix classification task exhibited a significant improvement in reading comprehension at post-test, which was not observed among groups receiving training in general flexibility or in a control task (dominoes).

In a later study, Cartwright et al. (2010) showed that general and reading-specific flexibility both improved between 1st and 2nd grades and that this improvement was not explained by increases in decoding ability. While each type of flexibility correlated with reading comprehension, reading-specific flexibility again proved to be a robust and independent predictor of reading comprehension among these younger children, whereas general flexibility contributed no additional variance beyond reading-specific flexibility. Altogether, Cartwright argues that this set of findings constitutes evidence that cognitive flexibility plays an important role in reading development, and further, that the component most crucial to progress is domain-specific.

Recently, Kieffer et al. (2013) found that flexibility in the Wisconsin Card Sorting Test correlated with reading comprehension but not with performance in a task measuring letter and word identification among their Grade 4 readers from low-income backgrounds. The results of path analyses indicated that flexibility was a significant and independent predictor of reading comprehension beyond the control variables (letter/word identification, language comprehension, working memory, processing speed, phonological awareness). Flexibility also made an indirect contribution to reading comprehension via language comprehension, which the authors interpreted as indicating that higher levels of flexibility may confer advantages in reading for meaning.

However, relations between flexibility and reading have proved more equivocal in other studies, especially those of reading acquisition in languages other than English. Monette et al. (2011) assessed flexibility among French-speaking kindergarteners’ with two tasks: a card sort task requiring a switch between two sorting rules and an adapted version of the Trail-making test (Trails P; Espy and Cwik, 2004). They found that flexibility failed to predict a composite measure of the children’s reading and writing skills in Grade 1. Although van der Sluis et al. (2007) did observe that flexibility scores from measures of task-switching efficiency were related to Dutch forth- and fifth-graders’ accuracy in a timed word reading task, the relationship found was negative.

Further exploration of this topic is clearly required given the failures to replicate evidence that cognitive flexibility is positively associated with reading progress. Our first objective is to determine whether the flexibility required in considering two dimensions simultaneously primarily applies to learning to read in opaque orthographies like English (Cartwright, 2002; Cartwright et al., 2010). Berninger and Nagy’s (2008) analysis points to a greater need for flexibility when mappings between the features of print are complex. Opaque orthographies have many-to-one or one-to-many mappings between orthography and phonology which slows the development of word reading (Seymour et al., 2003) and renders the activation of phonology from print difficult (Share, 2008). This may encourage beginning readers of English to make early use of the variety of information at their disposal (orthographic, phonological, semantic, contextual) and could account for the observed influence of reading-specific flexibility on reading comprehension (Cartwright et al., 2010). French has a more transparent system of grapheme–phoneme correspondences than English (Ziegler et al., 1996; Peereman et al., 2007; Moll et al., 2014), and French second-graders are known to make extensive use of phonological decoding in reading (Sprenger-Charolles et al., 2003). Hence, there may be less need for them to resort to other sources of information, raising the question of whether flexibility is critical for early reading comprehension in more transparent orthographies such as French.

A second, and related, objective is to test whether flexibility influences the reading of words in isolation as suggested by Berninger and Nagy (2008). Developmental models of reading comprehension give a central role to recognition of the written words that make up the sentences, paragraphs and text to be understood (Gough and Tunmer, 1986; Perfetti et al., 2005). Text reading comprehension is engaged by accessing the semantic code of words via visual recognition and the language processing mechanisms assemble these words into messages. The quality of access to word representations is critical within this framework and this dependence on the activation and manipulation of different codes (phonological, orthographic, semantic) makes it seem plausible that flexibility could play a role in this key aspect of reading comprehension. In our study, we attempt to answer this question with a single word reading task that requires activation not only of formal codes (phonological, orthographic) but also semantic codes. Our word reading task, therefore, allows examination of whether flexibility contributes to reading comprehension via the recognition of words in isolation and access to their meanings,

In relation to our third objective, an important question raised by Cartwright’s (2002, 2007) research bears on the domain-specificity of flexibility. Although most developmental research on flexibility does not consider the question of specificity, a few studies demonstrate that flexibility in matching tasks is highly dependent on the conceptual domain in question (Bialystok and Martin, 2004; Blaye et al., 2007; Maintenant and Blaye, 2008; Foy and Mann, 2013). While Cartwright’s results could be considered as support for this view, the contrast between her reading-specific and general flexibility tasks were not entirely conclusive. In the general cognitive flexibility task, participants had to sort line drawings of objects by color and by the superordinate category that the objects referred to, whereas in the reading-specific flexibility task, they had to sort words by their initial phoneme and by the superordinate category that the words referred to. That is, two potential sources of difference were confounded: the tasks differ both in terms of sorting criteria (perceptual/semantic versus phonological/semantic) and the kind of stimuli to which these criteria are applied (written words versus pictures). Hence, previous work remains inconclusive about which of the two features (stimuli versus criteria) is related to reading. To overcome this limitation, our study manipulates stimuli while keeping criteria equivalent (phonological/semantic).

In sum, the present study aims to investigate three important questions: (1) Is flexibility necessary in learning to read orthographies that are less opaque than English? (2) Does flexibility play a role in word reading as well as reading comprehension? and (3) Is the flexibility that is associated with reading, domain specific or domain-general?

## MATERIALS AND METHODS

### PARTICIPANTS

The participants were 60 second-graders (36 girls and 24 boys) from five schools with a middle-class catchment area in Aix-en-Provence in France (mean age: 7.63 years; SD = 0.30 years). In line with French Institutional and National regulations, four types of authorization were obtained for participation in this study: (1) written consent from the school authorities (the Inspector of National Education in France) in response to a written description of the research objectives and procedure of the study to be conducted with the child at school; (2) the consent of the head-teacher of the elementary school on the basis of information about the experimental procedure; (3) written informed consent from the child’s parents or guardians, in which it is explicitly explained that they can refuse to allow their child to participate without consequence for them or their child; and (4) children’s final enrollment was based on their own voluntary participation.

There were three additional inclusion criteria: (1) native speakers of French; (2) a reading level at least at chronological age on the French standardized test, “l’Alouette” (Lefavrais, 1967); and (3) non-verbal reasoning skills above the 25th percentile using the Raven’s Colored Progressive Matrices (PM47, Raven et al., 1995). The Alouette test is standardized for children aged from 5 to 14 years and involves reading aloud a text of 265 words as quickly and accurately as possible. The text contains real words in meaningless but grammatically correct sentences. Performance is converted into a reading age according to a standardized procedure taking account both of total reading time and accuracy.

### MATERIALS

#### Reading tasks ^3^

***Pseudo-word decoding.*** Sixty pseudo-words between 2 and 6 letters in length (e.g., pirda) were presented on a sheet of paper (10 pseudo-words per line). All were regular with regard to grapheme–phoneme correspondences but 20 contained graphemes whose pronunciation was context-dependent (i.e., s = /s/ or /z/; g = /g/ or /j/; c = /k/ or /s/). The number of pseudo-words read aloud correctly within one minute was recorded.

***Word reading.*** Both the recognition and comprehension of words was assessed by asking children to read a list of 108 words silently and to circle any animal names (*n* = 50). Items were selected from the 1000 most frequent words in Manulex (Lété et al., 2004) and distractors came from semantic categories such as fruits, vegetables, modes of transport, clothes, etc. (e.g., hibou, fusée, balai, loup, zèbre, tapis [English translation: owl, rocket, broom, wolf, zebra, carpet]). The word list was distributed across 18 lines of text (six words per line, each containing 2–4 animal names). Animal names increased in difficulty according to length and regularity of grapheme–phoneme correspondences. The number of animal names circled correctly within one minute was recorded. The error rate was negligible (*M* = 0.05; SD = 0.02).

***Passage reading comprehension.*** Performance was averaged across two tests. The first assessed the comprehension of short passages of text. Children read each sentence aloud and then traced a route on a map (e.g., Je vais du garage à la poste en passant par le parc [English translation: I go from the garage to the post office through the park]). Children could return to the text as often as they needed to. In the second task, children read aloud sentences referring to action sequences and then mimed what they had just read (e.g., Avec l’autre main, je prends le plus petit rond et je le mets sur le sol [English translation: With the other hand, I take the smallest circle and put it on the ground]). This test evaluated comprehension of anaphors (e.g., Je prends le grand carré avec une main et je le mets dans la boîte [English translation : I take the big square with one hand and I put it in the box]) and spatial terms (e.g., Je le pose ensuite entre les deux ronds puis sous la boîte [English translation : Next I put it between the two circles then under the box]). For each of these two tasks, a score was computed as a ratio of the number of correct actions to total time taken (in seconds).

#### Flexibility tasks

Two double classification tasks were derived from those used by Cartwright (2002), with the constraint of avoiding the potential confusion between the two types of differences that were present in the original versions of the tasks: (i) Word Flexibility – this was reading-specific as it involved the classification of printed words; and (ii) Picture Flexibility – this required classification of drawings and did not involve reading. Both tasks demanded the simultaneous processing of two dimensions: phonology and semantics. The experimenter first demonstrated the sorting of a set of 12 stimuli into a 4-cell matrix, explaining that sorting could be accomplished in two ways: According to what can be heard at the beginning of the picture name/word (phonological criterion) and according to the sorts of things the drawings/words referred to (semantic criterion). She then double-classified the 12 cards into the matrix, commenting on her performance: As you can see, I’m putting all the things starting with /p/ (pear, peach) into this row; and all the things starting with /b/into this row …. But look, in this column, I’m putting all the fruits … and in this one, I’m putting all the animals. Children then sorted five new sets of 12 cards and were asked to comment on each double classification.

Two points were awarded for each correct double classification with both criteria described verbally; 1 point for evidence of double classification in either card sorting or verbal justification; and 0 for any other performance^4^. Response time (in seconds) for each sorting trial was also computed. Performance was averaged across the five stimulus sets for each task and a flexibility score was computed as a ratio of accuracy to response time: (Acc/RT)^*^10.

### PROCEDURE

The children were tested in a quiet room within their schools over four sessions as follows: (1) Alouette reading, PM47; (2) word reading, passage reading comprehension; (3) word flexibility, pseudo-word decoding; and (4) picture flexibility. The order of the last two sessions was counterbalanced.

## RESULTS

**Table 1** describes participant characteristics and performance on the reading and cognitive flexibility tasks. Although *z*-scores are used in the regression analyses, untransformed scores are presented here for ease of interpretation. The children’s mean reading age (*M* = 94.65 months; SD = 7.34; Range = 85–119) was ahead of chronological age [*t*(59) = 2.89, *p* = 0.005].

**Table 1:** Pearson product-moment correlations, means, standard deviations and range for age, PM47 (raw scores), pseudo-word decoding, word reading, passage reading comprehension, picture and word flexibility scores (*N* = 60).

Measures	1	2	3	4	5	6	7
1. Chronological age	0.178	-0.134	-0.001	0.031	0.174	0.202
2. PM 47		-	0.059	0.101	0.243	0.020	0.204
3. Pseudo-word decoding			-	0.533^**^	0.482^**^	0.210	0.305^*^
4. Word reading				-	0.469^**^	0.325^*^	0.470^**^
5. Passage reading comprehension					-	0.293^*^	0.530^**^
6. Picture flexibility						-	0.642^**^
7. Word flexibility							-
*M*	91.55	29.07	39.15	15.95	23.10	0.56	0.67
SD	3.59	4.39	7.2	3.06	5.90	0.30	0.34
Range	84–100	16–35	22–53	8–22	13–41	0.07–2.08	0.16–1.90

*p < 0.05, two-tailed; **p < 0.01, two-tailed.

Correlations between variables are also reported in **Table 1**. As no significant correlations were observed involving chronological age or PM47, these variables were not entered in the final regression analyses. A preliminary series of regression analyses was also conducted, which established that inclusion of PM47 scores did not alter the pattern of results reported in the final analyses. Word flexibility scores correlated positively not only with word reading and passage reading comprehension but also with pseudo-word decoding; whereas picture flexibility scores did not correlate significantly with pseudo-word decoding, but showed a positive association with the two reading measures that involved the processing of meaning (word reading, passage reading comprehension).

Two hierarchical regression analyses were conducted with passage reading comprehension as the criterion variable. The traditional linguistic predictors, pseudo-word decoding and word reading were entered on the first two steps. In Analysis A (**Table 2A**), word flexibility scores were entered on the third step and picture flexibility on the fourth step. In Analysis B, the flexibility tasks were entered in the reverse order. Altogether these four variables accounted for nearly 40% of the variance in passage reading comprehension (**Table 2B**).

**Table 2:** Hierarchical regression analyses predicting passage reading comprehension with **(A)** word flexibility entered before picture flexibility; and **(B)** with picture flexibility entered before word flexibility.

	Predictors	Δ *R*^2^	β
**(A)**
Step 1	Pseudo-word decoding	0.232***	0.482
Step 2	Word reading	0.063*	0.297
Step 3	Word flexibility	0.110**	0.377
Step 4	Picture flexibility	0.005	-0.094
**(B)**
Step 1	Pseudo-word decoding	0.232***	0.482
Step 2	Word reading	0.063*	0.297
Step 3	Picture flexibility	0.018	0.144
Step 4	Word flexibility	0.097**	0.436

*p < 0.05, **p < 0.01, ***p < 0.001.

In each analysis, Word flexibility explained approximately 10% of the concurrent variance in passage reading comprehension over and above the more traditional linguistic predictors, and critically, after controlling for picture flexibility in Analysis B. In contrast, picture flexibility failed to explain any additional variance regardless of entry position.

Two new regression analyses were conducted with word reading as the criterion variable (**Tables 3A,B**). Decoding was entered as the first predictor, accounting for more than 28% of the variance. Picture flexibility contributed 4.8% of additional variance when entered before word flexibility, however, did not add any explanatory variance when entered after word flexibility. Word flexibility explained an additional 10.4% of the variance when entered before picture flexibility and 5.7% of the variance when entered on the final step; hence, confirming the critical role of the reading-specific, word flexibility task.

**Table 3:** Hierarchical regression analyses predicting word reading with **(A)** word flexibility entered before picture flexibility; and **(B)** picture flexibility entered before word flexibility.

	Variables	Δ *R*^2^	β
**(A)**
Step 1	Pseudo-word decoding	0.284***	0.533
Step 2	Word flexibility	0.104**	0.339
Step 3	Picture flexibility	0.000	0.029
**(B)**
Step 1	Pseudo-word decoding	0.284***	0.533
Step 2	Picture flexibility	0.048*	0.223
Step 3	Word flexibility	0.057*	0.321

*p < 0.05, **p < 0.01, ***p < 0.001.

## DISCUSSION

Our exploration of the concurrent relationship between cognitive flexibility and early reading had three main objectives: (1) to investigate whether flexibility is involved in learning to read an orthography that was more transparent than English, namely, the French orthography; (2) to examine the type of reading skills that are associated with flexibility, word reading and/or reading comprehension; and (3) to clarify whether domain-general or domain-specific cognitive flexibility mediates any such relationship with learning to read.

Our results show that reading acquisition in French is related to cognitive abilities that are not exclusively language-based. This extends Cartwright et al.’s (2010) findings from English to the French orthography. In other words, the flexible handling of orthographic, phonological, and semantic codes appears important even when reading a more transparent orthographic system. Word reading skills are acquired more rapidly in French (Seymour et al., 2003), which is thought to reflect a greater reliance on phonological decoding due to the level of consistency in the grapheme-to-phoneme mappings present in the orthography (Ziegler et al., 1996; Sprenger-Charolles et al., 2003). While it will be important to confirm this finding in orthographies with even higher levels of transparency such as Spanish or Finnish, our findings imply that flexibility has a role that extends beyond dealing with the complexities surrounding orthographic depth. This outcome is consistent with the growing number of studies that implicate cognitive abilities in reading acquisition (Conners, 2009; Sesma et al., 2009; Kendeou et al., 2014).

Our use of word reading as a predictor of passage reading comprehension allowed direct assessment of the consequences of the activation of semantic information about words during reading. Interestingly, word reading contributed more than 6% of the variance in passage comprehension beyond that contributed by pseudo-word decoding. This is consistent with Perfetti et al.’s (2005) hypothesis that reading comprehension is engaged by accessing the semantic code of words via visual recognition, and is supported by evidence that word meaning participates in single word reading from the initial phases of acquisition (Nation, 2008; Nation and Cocksey, 2009). Nevertheless, flexibility in coordinating phonological and semantic information made a contribution to the prediction of reading comprehension over and above the individual contribution of basic phonological and semantic processing skills. The critical influence of the simultaneous processing of dimensions is in keeping with the importance that has been placed on the coordination of multiple features of print in reading (Cartwright, 2002; Berninger and Nagy, 2008; Conners, 2009).

A novel and interesting result from our study is that flexibility predicts second grade reading for comprehension not only of texts but also of isolated words beyond the classic influence of decoding skills (e.g., Ouellette and Beers, 2010). A small but significant part of word reading was explained by general (picture) flexibility when it was entered before reading-specific (word) flexibility in the regression analysis. The picture flexibility task involves the coordinated use of phonological and semantic information about referents as does word reading. However, the reading-specific flexibility task, based on written words, still accounts for additional variance in word reading over and above general (picture) flexibility; whereas the reverse is not true. It was also reading-specific (word) flexibility rather than general (picture) flexibility that predicted passage reading comprehension beyond the influence of pseudo-word decoding and word reading. Together these findings support the interpretation that it is not only phonological-semantic rather than perceptual-semantic flexibility that operates in word and passage reading comprehension (Cartwright et al., 2010), but phonological-semantic flexibility in the specific context of written words.

In the present study, steps were taken to be precise about the nature of the link between cognitive flexibility and reading comprehension, especially in relation to the question of domain-specificity. In future work, it will be important to introduce controls for any non-executive demands that were imposed by the matrix classification tasks used as van der Sluis et al. (2004, 2007) have argued that the effects of any EF can only be fully understood after taking into account the implications of “task impurity.”

Indeed, the variety of tasks used to measure cognitive flexibility [see Introduction for a brief overview, and Diamond (2013) for a more thorough review], point to possible reasons for inconsistency in the findings regarding a role for flexibility in the development of reading skills. The task used to measure flexibility is one of the major differences between the present study and the other study of the French language by Monette et al. (2011). Monette et al. (2011) chose to use a card sort task and an adapted version of the Trail-making test, both tasks that require children to make a switch between two sorting criteria. This type of demand differs critically from the flexibility required by matrix classification tasks, such as those used in the present study, which require the simultaneous processing of two dimensions. Therefore, in line with the views of Cartwright (2002, 2007) and Berninger and Nagy (2008), our contention is that this simultaneous maintenance of two perspectives may be a critical component of developing reading skills due to the need to coordinate the multiple types of information contained in print.

Of course, in order to conclude that this task difference is critical, it will be important to rule out the influence of other differences between the two studies. Other differences include the reading measures used. Monette et al. (2011) employed a composite score based on word reading, spelling, and reading comprehension items from the French version of the WIAT-II administered in a group setting, whereas our reading tasks were administered individually and included the standardized Alouette reading test and separate assessments of specific literacy skills, namely word reading, passage reading comprehension, and decoding. Our intention was to obtain as accurate a picture as possible of the literacy skills that were related to flexibility and to exert control for other more well-known predictors of word reading and reading comprehension such as decoding ability; however, how far this objective was achieved remains to be established empirically.

As cognitive flexibility develops relatively late, our future work will include a longitudinal component to examine the coordination of phonological and semantic information in reading in relation to emerging flexibility at key points throughout preschool and elementary school, which should offer some causal insight into the role of flexibility in reading acquisition.

## CONCLUSION

Overall, these data contribute to the recent and rapidly growing field investigating the role of EF in reading acquisition. Flexibility in coordinating the processing of phonological and semantic information emerged here as a significant correlate of second grade word reading and passage reading comprehension in French. However, cognitive flexibility had greatest power as a predictor of comprehension, over and above traditional linguistic skills, when the matrix classification measures involved the manipulation of written words rather than pictures. Further research is required to explore our conclusion that the predictive value of this type of flexibility is a consequence of the need for an orthographic reading procedure that simultaneously generates phonological and semantic codes for subsequent processing to achieve comprehension.

## Conflict of Interest Statement

The authors declare that the research was conducted in the absence of any commercial or financial relationships that could be construed as a potential conflict of interest.

## Footnotes

1 However, see St Clair-Thompson and Gathercole (2006) for contrary evidence.

2 See Kloo and Perner (2003) and Kloo et al. (2010) for a related account in which flexibility is associated with the realization that a single object can be redescribed in a number of different ways.

3 The authors would like to express their gratitude to Liliane Sprenger-Charolles (personal communication) for generously allowing them to use her tests of word and non-word reading and passage-reading comprehension.

4 Cartwright’s (2002) procedure for item scoring is given here to ease comparison with her work : score = 3, child sorted correctly and provided a correct verbal justification; score = 2, child sorted incorrectly but provided a correct verbal justification for the Experimenter’s sort; score = 1, child sorted correctly but gave an incorrect (or no) verbal justification; and score = 0, child sorted incorrectly and gave an incorrect (or no) verbal justification. Note that the scoring system differs slightly in the present study because the Experimenter did not demonstrate the correct sort if a child made an error during the experimental trials.
